# The hanging chin sign as a mortality predictor in geriatric patients at the emergency department: a retrospective cohort study

**DOI:** 10.1186/s12877-022-02780-7

**Published:** 2022-02-03

**Authors:** Anneloes N. J. Huijgens, Laurens J. van Baardewijk, Carolina J. P. W. Keijsers

**Affiliations:** 1 Department of Geriatric Medicine, Jeroen Bosch Hospital, Henri Dunantstraat 1, 5223 GZ ‘s Hertogenbosch, the Netherlands; 2 Department of Radiology, Maxima Medisch Centrum, Postbus 7777, 5500 MB Veldhoven, the Netherlands

**Keywords:** Hanging chin sign, Chest X-ray, geriatric patients, mortality, emergency department

## Abstract

**Background:**

At the emergency department, there is a need for an instrument which is quick and easy to use to identify geriatric patients with the highest risk of mortality. The so- called ‘hanging chin sign’, meaning that the mandibula projects over one or more ribs on the chest X-ray, could be such an instrument. This study aims to investigate if the hanging chin sign is a predictor of mortality in geriatric patients admitted through the emergency department.

**Methods:**

We performed an observational retrospective cohort study in a Dutch teaching hospital. Patients of ≥65 years who were admitted to the geriatric ward following an emergency department visit were included. The primary outcome of this study was mortality. Secondary outcomes included the length of admission, discharge destination and the reliability compared to patient-related variables and the APOP screener.

**Results:**

Three hundred ninety-six patients were included in the analysis. Mean follow up was 300 days; 207 patients (52%) died during follow up. The hanging chin sign was present in 85 patients (21%). Patients with the hanging chin sign have a significantly higher mortality risk during admission (OR 2.94 (1.61 to 5.39), *p* < 0.001), within 30 days (OR 2.49 (1.44 to 4.31), *p* = 0.001), within 90 days (OR 2.16 (1.31 to 3.56), *p* = 0.002) and within end of follow up (OR 2.87 (1.70 to 4.84),p < 0.001). A chest X-ray without a PA view or lateral view was also associated with mortality. This technical detail of the chest x-ray and the hanging chin sign both showed a stronger association with mortality than patient-related variables or the APOP screener.

**Conclusions:**

The hanging chin sign and other details of the chest x-ray were strong predictors of mortality in geriatric patients presenting at the emergency department and admitted to the geriatric ward. Compared to other known predictors, they seem to do even better in predicting mortality.

**Supplementary Information:**

The online version contains supplementary material available at 10.1186/s12877-022-02780-7.

## Introduction

Worldwide, the number of older adults (aged 65 and over) presenting at the emergency department (ED) is increasing, particularly due to their growing proportion in the population [1–3]. In 2018–2019, 21% of all emergency department visits in the United Kingdom were made by patients aged 65 years and older [4]. In the Netherlands, this is even more, with patients aged 60–79 accounting for 29% of all ED visits [5]. Almost half (46%) of the ED visits among patients aged 70 and over resulted in a hospital admission [6]. Acute medical illness in hospitalised older adults is associated with functional decline and mortality during and after hospitalisation [7–9]. Within one year of acute admission at the geriatric ward, mortality rates are high, ranging from 17.2 to 40.2 worldwide [10].

There are several tools which predict mortality in the general population, but their value for the geriatric patient at the ED is still unclear. Risk assessment instruments for risk stratification of older adults following ED care do exist. However, Carpenter et al. [11] showed in a large systematic review and meta-analysis that none of the published risk stratification instruments (including ISAR, ISAR modifications, TRST and VIP) demonstrate sufficient prognostic accuracy to distinguish high-risk or low-risk subsets of geriatric patients in EDs. The authors demonstrated the same lack of prognostic accuracy for individual predictors of vulnerability and validated measurements of frailty. Recently, a screening tool for acute presenting older patients (APOP) was introduced that predicts adverse health outcomes for older ED patients. The investigators developed and validated prediction models for a 90-day composite outcome and 90-day mortality in older emergency patients [12, 13]. Presumably this is the best predictor of mortality in geriatric patients. Unfortunately, it is another new checklist added to the activities at the ED, which needs to be implemented.

Nonetheless, it is important to estimate the prognosis for the geriatric patient. Identification of those at highest risk presents the opportunity to offer the correct intensity of care and to guide preventive interventions and informed decisions about treatment [14]. Therefore, there is a need for a quick and easy-to-use tool to predict mortality in the geriatric patient.

In clinical practice, there is a subjective feeling that the ‘hanging chin sign’ is related to a worse outcome. This sign can be observed on the chest X-ray, and is present when the mandibular bone is projected over one or more ribs [ 15]. It is an objective sign which is easy to interpret. The hanging chin is also seen in patients with lordosis or kyphosis or neuromuscular disorders [15]. In practice, it is often difficult to differentiate. However, different studies have shown that kyphosis is also associated with a poor prognosis [16–18]. This makes the differentiation less relevant. Van Beijnen et al. recently showed that critically ill patients consulted by an intensive care unit (ICU) physician at the ED and displaying a hanging chin have higher in-hospital mortality [15]. But further evidence is lacking, especially for the geriatric patient admitted at the general geriatric ward following the ED visit.

Furthermore, there is a subjective feeling that the lack of a posterior (PA) view (i.e. presence of an AP view) and lack of a lateral view of the chest X-ray are related to a worse outcome as well. When absent, this suggests that it was not possible to stand or to sit straight and raise the arms, respectively.

Our hypothesis is that the inability of keeping the head up, being unable to stand or raising up the arms, is an expression of severe illness and /or lack of resilience and/or frailty. Since these three factors are predictors of mortality [19–22], we hypothesize that the hanging chin sign and the absence of PA view and lateral view are predictors of mortality as well.

This study aims to assess if the sign of a hanging chin is a predictor of mortality in geriatric patients admitted through the ED. Secondly, the study aims to investigate if technical details related to the X-ray (e.g. presence of PA, AP or lateral view) are also predictors of mortality. Other secondary objectives are to assess the association between the hanging chin sign, the length of hospital admission and the destination of discharge, and to compare the hanging chin sign with the APOP screening tool.

## Methods

### Study design & participants

A single-centre, observational retrospective cohort study was performed in a large teaching hospital in the Netherlands. Data were extracted from the electronic health record and were part of standard care. Patients who were admitted to the geriatric ward following an ED visit between January 1, 2018 and December 31, 2018 were included. Additional inclusion criteria were: being aged 65 years or older and having had a chest X-ray performed at admission. The first admission was selected when patients were admitted more than once during the enrolment period. Exclusion criteria were:

- hip fracture as admission diagnosis, because although these patients were admitted to the orthopedic ward and their condition, hospitalisation and prognosis take a different course.
- outlying patients, which means that the patient was admitted to be seen by the geriatrician but stayed on another ward, and therefore did not receive standard geriatric care
- transfers from other hospitals, because these patients were seen at a later stage in course of their illness

The follow-up ended on November 5, 2019.

### Automatic data extraction using CTcue

Patients were selected using CTcue, an application which structures and collects data from the electronic health record system and is widely used in Dutch and Belgium hospitals [23]. All patients with an age ≥ 65 and admitted to the geriatric ward during the study period were extracted. All extracted patients were then manually verified in the electronic health record, using inclusion and exclusion criteria. Doubles were checked. Age, sex, admission and discharge dates were obtained from CTcue, other data was obtained from chart review. The collected data were standard elements of the comprehensive geriatric assessment performed on every patient admitted through the ED. The data were collected using a system with predefined criteria for classifying recorded data. Patients and families were not approached for any additional data.

### Patient characteristics

Patient characteristics were selected based on their use in assessing frailty in daily practice and previous research [11, 12]. Patient characteristics were divided in general characteristics, characteristics of the ED visit and geriatric features.

First, general patient characteristics were collected: age, sex, admission and discharge dates and admission diagnosis. In some patients there were multiple admission diagnoses; in these cases, we selected the one that was mentioned first in the conclusion of the admission letter.

Second, patient characteristics related to the ED visit and geriatric features were collected: mode of transportation to the ED, living independently (including sheltered home or living with relatives) or institutionalised, number of medications at admission (where a fixed dose combination was counted as 1), polypharmacy (≥5 medicine prescribed), help needed during bathing/showering and dressing, help needed during daily activities, previous hospital admission in the past six months, fall-related ED visit, the need for laboratory testing, presence of diagnosis of ‘dementia’ and the two questions ‘in which year do we live?’ and ‘in which month do we live?’ to assess orientation in time.

### Chest X-ray

All chest X-rays were retrospectively reviewed. The following details were documented: presence of the hanging chin sign, patient position of acquisition (standing, sitting or lying), projection view (Posteroanterior (PA) or anteroposterior (AP)) and presence of a lateral view. In case of doubt, an independent blinded radiologist (LB) was consulted. Disagreement was resolved by consensus between the researcher AH and radiologist LB.

**Fig. 1:**
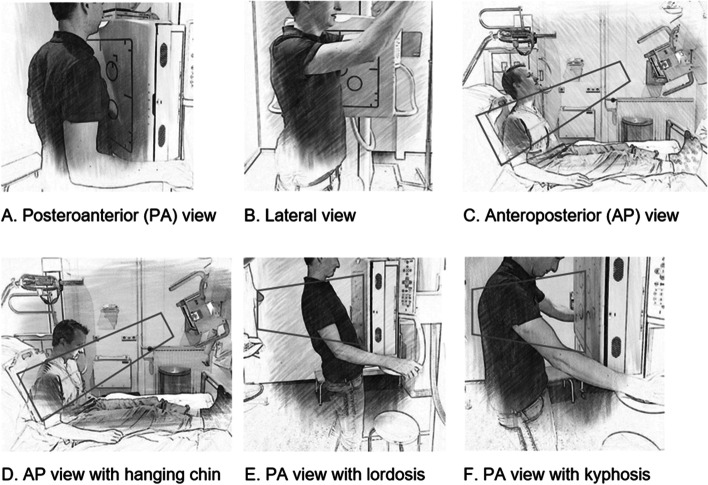
Projection views of chest X-ray (Figure by author). **A** and **B** show the standard projections of the chest X-ray. The patient is standing against the detector. **C** shows an AP projection without the presence of a hanging chin sign. **D**, **E** and **F** show examples in which a hanging chin sign could be seen on the chest X-ray

Figure 1 shows the different projection views of the chest X-ray (1A-C). Preferably, a chest X-ray consists of a standing PA view and lateral view. When a patient is unable to stand, an AP view is acquired in sitting or even lying position. An AP view is non-preferable because of lower quality and magnification of the heart, making it more difficult to evaluate [24].

The hanging chin sign is most commonly seen on a chest X-ray made in AP view (Fig. 1D). The hanging chin is also seen in PA view, but to a lesser extent, especially in patients with lordosis or (hyper) kyphosis (Fig. 1E-F). Neuromuscular disorders are also mentioned as a cause [25].

### Hanging chin sign

**Fig. 2:**
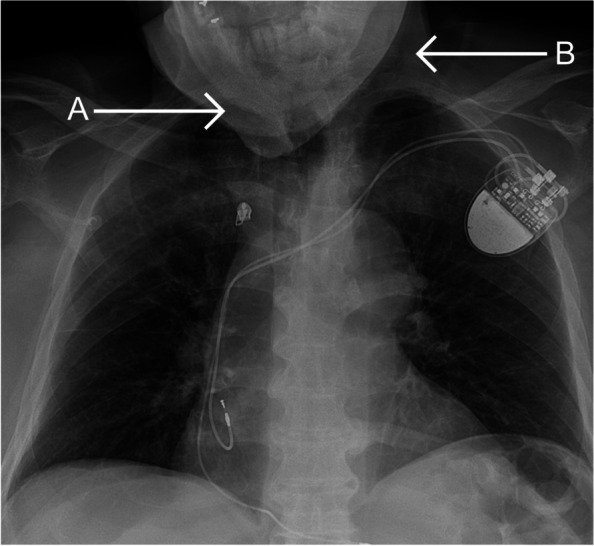
Hanging chin sign: mandibular bone (A) projected over the first rib (B)

The ‘hanging chin sign’ on the chest X-ray was considered positive when the mandibular bone was projected over one or more ribs (Fig. 2) [15]. Patients were excluded when the chest X-ray was incomplete or was of poor quality.

### Primary outcome measures

Primary outcome was mortality during admission, within 30 days, within 90 days and at the end of follow up. Mortality was obtained from the electronic health record and double- checked in the national mortality registry.

### Secondary outcome measures

Details of the chest X-ray were obtained from the electronic health record. Projection view (posteroanterior (PA) or anteroposterior (AP)) and patient position of acquisition (standing, sitting or lying) were displayed on the chest X-ray itself or included in the report. The discharge destination was obtained from the electronic health record. The different discharge destinations were divided into four categories: 1. Home, sheltered home or living with children, 2. Nursing home or psychiatric institution, 3. Geriatric revalidation care and 4. Hospice or terminal care. We compared the residency before admission with the discharge destination and reported whether there was any change after admission. The admission length was calculated using the dates of admission and discharge.

According to the formula of the APOP study, a 90-day mortality risk was calculated [12]. Subsequently, patients were divided into high- and low-risk groups as in the APOP study.

Patients end up in the high risk group, when they belong to the 20% of patients with the highest APOP score. In our study, this corresponds to a APOP score of 45% or higher.

### Data analysis

Data were transferred to the Statistical Package for Social Sciences (SPSS, IBM) version 22 for analysis.

Outcomes between patients with and without a hanging chin sign were compared using the χ2 test and logistic regression, calculating Odds ratios and Kaplan-Meier curves. Sensitivity, specificity, positive likelihood ratio and negative likelihood ratio were calculated for the hanging chin sign and APOP high risk. Confidence intervals for the likelihood ratios were calculated using the “Log method” [26]. The hanging chin sign and APOP high risk were compared using likelihood ratios and confidence intervals: there is a significant difference when the LRs of the hanging chin sign fall outside the confidence intervals of the APOP high risk.

Patient-related variables (age, number of medications, polypharmacy, way of living, arrival by ambulance, help needed with bathing/dressing, fall related visit) have all been analysed for prediction of mortality, because each component is used separately in daily practice while assessing frailty. χ2 Test or logistic regression were used and Odds ratios were calculated. Outcomes of the APOP screening tool were analysed for prediction of mortality by using the χ2 test or logistic regression and calculating Odds ratios.

Length of hospital stay was tested for normality by Kolmogorov-Smirnov and Shapiro-Wilk tests. Outcomes were compared using the Mann-Whitney test. Discharge location and change in discharge destination were analysed using the χ2 test.

A sample size calculation was not performed, due to the lack of incidence rates in the literature. We assumed that a sample of this size should be sufficient to demonstrate if the ‘hanging chin sign’ is a suitable predictor of mortality or not.

## Results

### Patient selection

**Fig. 3:**
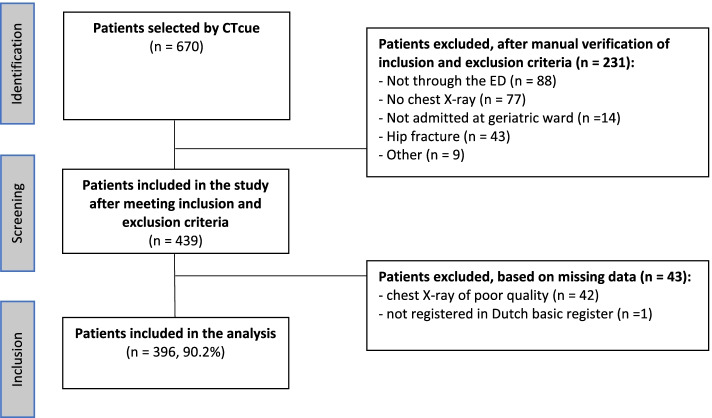
Flow diagram of patient selection. Abbreviation: *ED* emergency department

As shown in Fig. 3, CTcue extracted 670 patients. After manual verification, 439 patients met the inclusion criteria.

Data extraction was performed for these 439 patients. By doing this, 42 patients were excluded because of poor quality of the chest X-ray which meant that we were unable to identify the first rib, and one patient was excluded because they were lost to follow up, due to absence of registration in the Dutch basic register. Therefore, 396 patients of 439 patients (90.2%) who met the inclusion criteria were included in the analysis. There were no missing data, except for orientation in time and year (both available in only 215 patients) and for patient position of the AP view (available in only 264 patients).

### Patient characteristics

A total of 396 patients were included. As shown in Table 1, the mean age of the total study population was 85.1 years (standard deviation (SD) 6.4 years; range 65–101 years). Of the patients, 224 (57%) were female. The mean follow-up was 300 days (SD 220 days), with a maximum of 672 days. At the end of the follow-up, 207 patients (52%) had died. Table 2 shows mortality numbers per endpoint. The hanging chin sign was present in 85 patients (21%). Patients with evidence of the hanging chin sign were significantly more likely to live in residential care or a nursing home (*p* = 0.001), arrived more often by ambulance (*p* = 0.001), needed more help with bathing or dressing (*p* = 0.011) and had a higher APOP 90-day mortality score, compared with patients without the hanging chin sign. Other characteristics were not significantly different between the two groups.

**Table 1:** Patients characteristics

Characteristics	All patients	No hanging chin sign (*n* = 311)	Hanging chin sign (*n* = 85)	Statistics
Demographics				
Age (SD)	85.1 (SD 6.7)	85.0 (SD 6.7)	85.3 (SD 6.7)	*p* = 0.984
Female (%)	224 (56.6%)	176 (56.6%)	48 (56.5%)	*p* = 0.984
Living in residential care or nursing home (%)	81 (20.5%)	53 (17.0%)	28 (32.9%)	***p*** **= 0.001**
ED visit				
Arrival by ambulance (%)	302 (76.3%)	226 (72.7%)	76 (89.4%)	***p*** **= 0.001**
Fall-related ED visit (%)	92 (23.2%)	73 (23.5%)	19 (22.4%)	*p* = 0.828
Diagnosis at admission (%)				*p* = 0.444
* Infectious disease	230 (58.1%)	176 (56.6%)	54 (63.5%)	
* Heart disease	23 (5.8%)	18 (5.8%)	5 (5.9%)	
* Delirium	42 (10.6%)	31 (10.0%)	11 (12.9%)	
* Falling/mobility disorder	47 (11.9%)	40 (12.9%)	7 (8.2%)	
* Other	54 (13.6%)	46 (14.8%)	8 (9.4%)	
Indication for laboratory testing (%)	396 (100%)	311 (100%)	85 (100%)	–
Geriatric measurements				
Hospital admission in past six months (%)	81 (20.5%)	65 (20.9%)	16 (18.8%)	*p* = 0.674
Number of different medications (SD)	8.6 (SD 4.3)	8.2 (SD 4.1)	10.0 (SD 4.6)	*p* = 0.125
Help needed with bathing/dressing (%)	277 (69.9%)	208 (66.9%)	69 (81.2%)	***p*** **= 0.011**
Help needed with daily activities (%)	359 (90.7%)	279 (89.7%)	79 (92.9%)	*p* = 0.077
Disorientated in year (*N* = 215) (%)	125 (58.1%)	100 (59.2%)	25 (54.3%)	*p* = 0.557
Disorientated in month (*N* = 215) (%)	109 (50.7%)	88 (52.1%)	21 (45.7%)	*p* = 0.440
History of dementia (%)	144 (36.4%)	109 (35.0%)	35 (41.2%)	*p* = 0.298
APOP score				
APOP risk score (90-day mortality) (SD)	26.6 (SD 19.4)	25.2 (SD 18.3)	31.8 (SD 22.5)	***p*** **= 0.001**
APOP high risk (%)	77 (19.4%)	51 (16.4%)	26 (30.6%)	***p*** **= 0.003**

Abbreviations: SD, standard deviation; ED, emergency department; APOP, acute presenting older patient

**Table 2:** Mortality numbers per endpoint

	Mortality during admission n, (%)	Mortality at 30 days n, (%)	Mortality at t 90 days n, (%)	Mortality at t the end of follow up (mean 300 days) n, (%)
Total (*n* = 396)	55 (14%)	76 (19%)	115 (29%)	207 (52%)
- patients with hanging chin sign (*n* = 85)	22 (26%)	27 (32%)	36 (42%)	61 (72%)
- patients without hanging chin sign (*n* = 311)	33 (11%)	39 (16%)	79 (25%)	146 (47%)

The AP view of the chest X-ray was present in 288 patients (73%). In 76 patients (19%), both the hanging chin sign and AP view were present. The majority of the patients with the hanging chin sign had an AP view as well (89% patients). The supplementary table shows the patients characteristics at baseline of the patients with hanging chin sign and/or AP view.

### Primary outcome

Table 3 shows that patients with the hanging chin sign have a significantly greater risk of dying during admission (OR 2.942 (1.607–5.386), *p* < 0.001), within 30 days (OR 2.489 (1.437–4.311), *p* = 0.001), within 90 days (OR 2.158 (1.308–3.558), *p* = 0.002) and within the end of follow-up (OR 2.872 (1.704–4.842), *p* < 0.001).

**Table 3:** Variables that can predict mortality

	Death during admission		30-day mortality		90-day mortality		Death (mean follow up 300 days)	
**Chest X-ray**	OR (95% CI)	*p*	OR (95% CI)	*p*	OR (95% CI)	*p*	OR (95% CI)	*p*
Hanging chin sign	2.94 (1.61–5.39)	**< 0.001**	2.49 (1.44–4.31)	**0.001**	2.16 (1.31–3.56)	**0.002**	2.87 (1.70–4.84)	**< 0.001**
AP-view instead of PA-view	4.33 (1.68–11.17)	**0.001**	3.34 (1.60–6.96)	**0.001**	2.17 (1.26–3.73)	**0.005**	1.62 (1.04–2.53)	**0.033**
Lateral view absent	2.31 (1.28–4.17)	**0.005**	1.87 (1.10–3.19)	**0.020**	1.92 (1.19–3.08)	**0.007**	1.28 (0.81–2.00)	0.289
**Patient related variables**	OR (95% CI)	*p*	OR (95% CI)	*p*	OR (95% CI)	*p*	OR (95% CI)	*p*
Age	1.04 (0.99–1.08)	0.119	1.03 (0.99–1.07)	0.147	1.05 (1.01–1.09)	**0.006**	1.05 (1.01–1.08)	**0.004**
Number of medications	1.05 (0.99–1.13)	0.117	1.06 (1.00–1.13)	**0.039**	1.05 (0.99–1.10)	0.076	1.08 (1.03–1.13)	**0.002**
Polypharmacy	0.59 (0.30–1.15)	0.117	1.07 (0.55–2.08)	0.835	1.05 (0.60–1.86)	0.858	1.33 (0.79–2.22)	0.281
Living institutionalised	1.10 (0.51–2.20)	0.787	1.52 (0.85–2.71)	0.159	1.29 (0.76–2.18)	0.340	1.85 (1.12–3.07)	**0.016**
Arrival by ambulance	1.47 (0.71–3.05)	0.297	1.48 (0.78–2.78)	0.226	1.26 (0.74–2.13)	0.391	1.07 (0.67–1.69)	0.788
Help needed with bathing/dressing	1.46 (0.75–2.83)	0.264	1.62 (0.90–2.93)	0.104	2.08 (1.24–3.49)	**0.005**	2.20 (1.42–3.42)	**< 0.001**
Fall-related emergency department visit	1.15 (0.60–2.22)	0.674	1.13 (0.63–2.02)	0.685	0.95 (0.57–1.60)	0.851	0.89 (0.56–1.42	0.618
**APOP score**	OR (95% CI)	*p*	OR (95% CI)	*p*	OR (95% CI)	*p*	OR (95% CI)	*p*
APOP 90-day mortality risk (%)	1.01 (0.99–1.02)	0.245	1.01 (1.00–1.03)	**0.049**	1.02 (1.01–1.03)	**0.002**	1.03 (1.01–1.04)	**< 0.001**
APOP 90-day high vs low risk	1.19 (0.59–2.37)	0.632	1.25 (0.68–2.29)	0.474	1.52 (0.90–2.58)	0.115	1.91 (1.14–3.20)	**0.013**

Abbreviations: OR, odds ratio; CI, confidence interval; AP, anteroposterior; PA, posteroanterior; APOP score, acute presenting older patients score [8]

Sensitivity, specificity, positive likelihood ratio and negative likelihood ratio of the hanging chin sign for death during admission, within 30 days, within 90 days and at the end of follow-up are shown in Table 4. Positive likelihood ratios range from 1.80 to 2.32, negative likelihood ratios from 0.74 to 0.83.

**Table 4:** Sensitivity, specificity, positive likelihood ratio and negative likelihood ratio of the hanging chin sign and APOP score high risk

	Sensitivity, %	Specificity, %	Positive LR (CI)	Negative LR (CI)
**Hanging chin sign**				
Death during admission	40.0	81.5	2.16 (1.46–3.21)	0.74 (0.59–0.92)
30-day mortality	35.5	81.9	1.96 (1.34–2.87)	0.79 (0.66–0.94)
90-day mortality	31.3	82.6	1.80 (1.24–2.60)	0.83 (0.73–0.95)
Death at the end of follow up (mean 300 days)	29.5	87.3	2.32 (1.51–3.56)	0.81 (0.73–0.90)
**APOP score high risk**				
Death during admission	21.8	80.9	1.14 (0.66–1.98)	0.97 (0.83–1.12)
30-day mortality	22.4	81.3	1.19 (0.74–1.92)	0.96 (0.84–1.09)
90-day mortality	24.4	82.6	1.40 (0.93–2.11)	0.92 (0.82–1.03)
Death at the end of follow up (mean 300 days)	24.2	85.7	1.69 (1.11–2.59)	0.88 (0.80–0.97)

Abbreviations: *LR* likelihood ratio, *CI* confident intervals

**Fig. 4:**
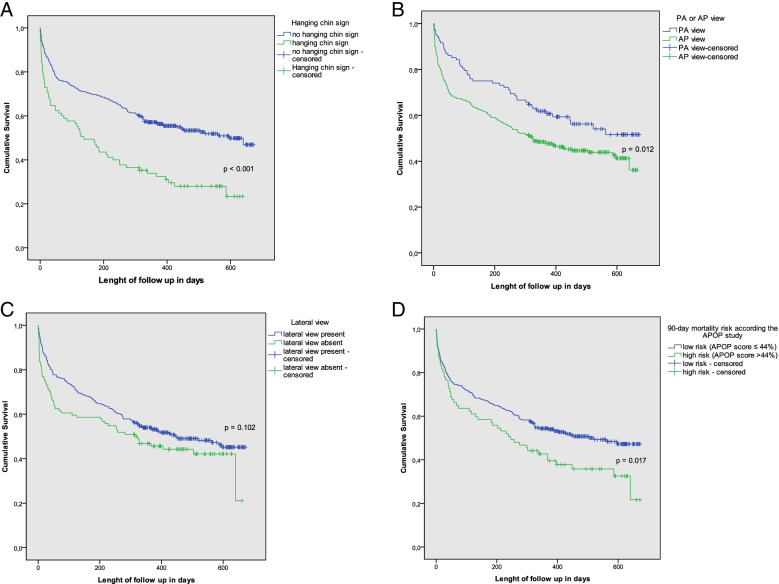
Kaplan-Meier plot of estimated survival in patients with. **A**. Hanging chin sign vs. no hanging chin sign, **B**. PA-view vs. AP-view, **C**. Lateral view present vs. lateral view absent, **D**. 90-day mortality risk according the APOP study, high risk vs. low risk. Abbreviations: *PA* posteroanterior, *AP* anteroposterior, *APOP* acute presenting older patient [8]

Figure 4 shows the Kaplan-Meier survival curves for the length of time after admission until death or end of follow-up. There was a significant difference in survival time between patients with the hanging chin sign and patients without the hanging chin sign (*p* < 0.001) (Fig. 4A). The mean survival in patients with hanging chin sign was 199 days (e.g. 6.6 months) vs. 383 days (e.g. 12.8 months) in patients without hanging chin sign.

### Secondary outcomes

Table 3 shows the variables that were tested for their ability to predict mortality: aspects of chest X-ray, patients related variables and the APOP score. The different aspects of the chest X-ray are also significant individual predictors of death during admission, 30-day mortality, 90-day mortality and death at the end of follow-up (mean 300 days). Patient-related variables are less able to predict mortality, especially when it comes to death during admission and 30-day mortality. As shown in Table 3, the hanging chin sign and the presence of an AP view are the only variables that are significant on all outcomes.

Figures 4B shows that there was a significant difference in survival time between patients with AP view and PA view, in favour of the PA view (log rank test *p* = 0.012). The presence of a lateral view seems to predict a better 30-day and 90-day survival, but was not significant in the long term (Fig. 4C).

The APOP score was also capable of predicting mortality, except for death during admission. The cut-off point for the high-risk group in our study was a APOP risk score of 44.3%. As shown in Table 3, high risk according to the APOP score was not a predictor of death during admission (*p* = 0.632), 30-day mortality (*p* = 0.474) and 90-day mortality (*p* = 0.115), but it was of death till the end of follow-up (*p* = 0.013). Table 4 shows the sensitivity, specificity, positive likelihood ratio and negative likelihood ratio of the APOP high risk for death during admission, within 30 days, within 90 days and at the end of follow-up. The likelihood ratios of the hanging chin sign for death during admission and 30-day mortality are significantly better than the likelihood ratios of the APOP high risk (e.g. the LRs of the hanging chin sign do not fall within the confidence intervals of the LR of the APOP high risk). This does not apply for 90-day mortality and death till end of follow up. Figure 4D shows the Kaplan-Meier plots of APOP high vs. low risk.

Length of admission was not significantly different (*p* = 0.852) between patients with the hanging chin sign (mean 12.1 days, SD 15.6 days) and patients without the hanging chin sign (mean 10.3 days, SD 10.9 days). After exclusion of patients who died during admission, length of admission was still not significantly different (*p* = 0.239).

Table 5 shows the discharge destination of the 341 patients (86%) who survived hospital admission. Although patients with the hanging chin sign were more likely to go to a nursing home or psychiatric institution, there was no significant difference (p = 0.239) in discharge destinations between patients with and without hanging chin sign. Furthermore, there was no significant difference regarding change in discharge destination between patients with and without hanging chin sign (OR 1.047 (CI = 0.543 2.018), *p* = 0.892); resp. 22.2% (14/63) and 23% (64/278) of the patients were not able to go to their previous living place and needed more intensive or terminal care.

**Table 5:** Discharge destination

	Discharge destination				Total
	Home/sheltered/with children	Nursing home/psychiatric institution	Geriatric revalidation care	Hospice/terminal care	
**No hanging chin sign**	171 (61.5%)	85 (30.6%)	14 (5.0%)	8 (2.9%)	278 (100%)
**Hanging chin sign**	30 (47.6%)	26 (41.3%)	4 (6.3%)	3 (4.8%)	63 (100%)
**Total**	201 (58.9%)	111 (32.6%)	18 (5.3%)	11 (3.2%)	341 (100%)

## Discussion

This study shows that the hanging chin sign is a predictor of mortality in geriatric patients admitted at the geriatric ward through the ED, in both the short and long term. This confirms our hypothesis that the hanging chin sign in geriatric patients is associated with a poor prognosis. In our study, this predictor is a stronger and/or more consistent predictor than known predictors such as age, help needed with bathing/dressing (i.e.. activities of daily living) and institutionalization. Our study also shows that the hanging chin sign is significantly better than the APOP in predicting death during admission and 30-day mortality. Predictive performance of the hanging chin sign was also compared with the predictive performance of other screening instruments, as reported by Carpenter et al.: positive and negative likelihood ratios of the hanging chin sign appeared to be mostly better than those of the ISAR, TRST and other (frailty) screening instruments [11]. Other predictors of mortality deriving from the chest X-ray were the presence of an AP view (instead of a PA view) and the absence of a lateral view. Both the hanging chin sign and other chest X-ray features are objective and easily available markers, which makes the chest X-ray a quick and easy-to-use tool to predict mortality in the geriatric patient. Our findings suggest that the above-mentioned features of the chest X-ray are able to identify the patients with severe illness and/or frailty and thus poor prognosis. Another explanation might be that the radiodiagnostic technicians assess the patient as to weak for a PA view, and thereby increase the risk of the hanging chin sign.

This study is the first to investigate if the hanging chin sign is a predictor of mortality in geriatric patients admitted to the geriatric ward. Although the hanging chin sign is something which is often referred to in Dutch hospitals, Van Beijnen et al. were the first and only ones to describe this sign [15]. They showed that critically ill adult patients presenting at the ED with the hanging chin sign have a higher in-hospital mortality risk. Our study showed that the hanging chin sign is also applicable to the geriatric patient presenting at the ED and admitted to the geriatric ward, and is also useful for predicting 30-day, 90-day and 300-day mortality. We found no association with change in discharge destination or length of admission, nor did Van Beijnen et al. for length of admission.

Baseline characteristics did differ slightly between the two groups. This suggests that the patients with the hanging chin sign were more frail and/or ill at baseline than patients without the hanging chin sign. The same applies to patients with an chest X-ray with AP view instead of PA view. However, we believe that is a logical consequence: the presence of the hanging chin sign as well as an AP view is a reflector of severity of frailty and illness. Correcting for this would devaluate the hanging chin sign because it removes the cause of the sign. Therefore, multivariate logistic and linear regression analysis was not performed either. We believe that the hanging chin sign incorporates all the other factors. It would be interesting to investigate which factor has the greatest influence on developing a hanging chin: frailty, resilience or severity of illness. Modification of age might be a factor as well.

There are some limitations to this study. First, we performed a retrospective study using data from the electronic health record. This makes it difficult to recall missing data and interpret some results. The chart abstractor was not blinded for the study hypothesis. We tried to minimise the effect of this by using clear predefined criteria, leaving very little interpretation. Furthermore, a blinded radiologist was consulted when there was doubt about the presence of the hanging chin sign.

Second, we included only the geriatric patients at the emergency department that underwent an X-ray and were admitted to the geriatric ward. In the Netherlands, only patients with complex comorbidities, an unclear primary organ problem, cognitive problems and/or interference on the social and/or functional domain are admitted to the geriatric ward. This corresponds to 17.6% of all the patients aged 70 or older presenting at our ED. The mortality rate in our cohort was 52%, which is higher than previous reported numbers [10]. Probably, this is explained by the above mentioned selection criteria, mean age of 85 years and by the fact that 58% of the patients was diagnosed with an infection. Therefore, our cohort consisted of a selected population. This causes selection bias and could limit the generalizability.

In our hospital, about 90% of the geriatric patients presenting at the emergency department was admitted. Patients that were discharged from the emergency department were not included in this study. Although not supported with numbers, we have a feeling that the patients with a hanging chin sign will be underrepresented in this group.

Third, 42 patients (9.6%) were excluded because of the poor quality of the chest X-ray. The quality of these X-rays was poor because the upper part of the thorax (e.g. upper ribs) was missing or shown unclear (e.g. due to overprojection of clothes or obesity). Therefore it was not possible to see if the mandibular bone was projected over the first rib. For comparison, Van Beijnen et al. [15] excluded 26% of patients for this reason.

Finally, in this study the 90-day *mortality risk* according to the APOP study was used. However, the APOP study group only validated the 90-day *composite outcome.* The 90-day mortality risk formula was composed to calculate a 90-day mortality risk, not death during admission, 30-day mortality or 300-day mortality. Furthermore, this screener is validated for every older patient at the ED, not only for those who were admitted to the geriatric ward. But despite all this, the cut-off point in our study for high vs. low risk (score of 44.3% or higher) was comparable to the cut-off point of the APOP study itself, suggesting that the study population of both trials were comparable [12, 13]. However, this would be an interesting subject of future research.

The results of our study suggest that the standard chest X-ray contains more information than one might think. The chest X-ray gives easily interpretable information of mortality in an early stage, in addition to history taking and physical examination. It could help identify those at risk of mortality, which might allow optimizing therapy for each patient. For example, treatment can be intensified. Furthermore, our results can be helpful in estimating the course of disease and prognosis, which can be used in the conversation with the patient and/or family.

## Conclusions

In conclusion, in this study, the hanging chin was a rather strong predictor of mortality in geriatric patients presenting at the ED and admitted to the geriatric ward. Other hidden features of the chest X-ray (absence of PA view and/or lateral view) were also predictors of mortality in these patients. Therefore, the chest X-ray can be helpful in identifying those with a poor prognosis.

This study is conducted in a selected population, which limits the generalizability.

Future research should focus on the predictive value of the hanging chin sign in all patients aged 65 and older at the ED. It should be of prospective design and focus on predicting functional decline as well. Furthermore, it would be interesting to study the cause of the hanging chin sign.

## Supplementary Information

**Additional file 1.**

## Data Availability

The datasets used and/or analysed during the current study are available from the corresponding author on reasonable request.

## Abbrevations

ED: Emergency department
ISAR: Identifications of seniors at risk (screening tool)
TRST: Triage risk screening tool
VIP: Variable Indicative of Placement risk (screening tool)
APOP: Acute presenting older patients (screening tool)
ICU: Intensive care Unit
PA: Posteroanterior
AP: Anteroposterior
SPSS: Statistical Package for Social Sciences
WMO: Law on Medical Research (*Wet medisch-wetenschappelijk onderzoek*)
WGBO: Medical Treatment Contracts Act (*Wet op de geneeskundige behandelingsovereenkomst)*
SD: Standard deviation
OR: Odds ratio
CI: Confidence interval
Resp.: Respectively
