# Supplementary material for: The hanging chin sign as a mortality predictor in geriatric patients at the emergency department: a retrospective cohort study
Source: BMC Geriatr. 2022 Feb 3;22:95. doi: 10.1186/s12877-022-02780-7 (PMC8815262; doi:10.1186/s12877-022-02780-7)
Supplement: Supplementary file 1 — Additional file 1. [file 12877_2022_2780_MOESM1_ESM.docx]

*Supplementary table. Patients characteristics at baseline, comparing patients with hanging chin sign and/or AP view.*

| **Characteristics** | **No hanging chin sign or AP view (n = 99)** | **Only hanging chin sign (n = 9)** | **Only AP view**  **(n = 212)** | **Both hanging chin sign and AP view (n=76)** | **Statistics** |
| --- | --- | --- | --- | --- | --- |
| Demographics | | | | | |
| Age | 83.5 (SD 7.0) | 85.3 (SD 5.6) | 85.7 (SD 6.4) | 85.4 (SD 6.8) | p = 0.065 |
| Female | 63 (63.6%) | 6 (66.7%) | 113 (53.3%) | 42 (55.3%) | p = 0.399 |
| Living in residential care or nursing home | 9 (9.1%) | 1 (11.1%) | 44 (20.8%) | 27 (35.5%) | p < **0.001** |
| ED visit | | | | | |
| Arrival by ambulance | 41 (41.4%) | 6 (66.7%) | 185 (87.3%) | 70 (92.1%) | p < **0.001** |
| Fall-related ED visit | 19 (19.2%) | 2 (22.2%) | 54 (25.5%) | 17 (22.4%) | p = 0.673 |
| Geriatric measurements | | | | | |
| Hospital admission in past six months | 20 (20.2%) | 1 (11.1%) | 45 (21.2%) | 15 (19.7%) | p = 0.899 |
| Number of different medications | 7.4 (SD 3.9) | 10.0 (SD 5.0) | 8.5 (SD 4.2) | 10.0 (SD 4.5) | p = **0.001** |
| Help needed with bathing/dressing | 49 (49.5%) | 5 (55.6%) | 159 (75%) | 64 (84.2%) | p < **0.001** |
| Help needed with daily activities | 82 (82.8%) | 9 (100%) | 197 (92.9%) | 70 (92.1%) | p = **0.027** |
| History of dementia | 30 (30.3%) | 4 (44.4%) | 79 (37.3%) | 31 (40.8%) | 0.468 |
